# A retrospective analysis of real-world height outcomes of growth hormone treatment in Syrian children

**DOI:** 10.1186/s12902-025-02116-7

**Published:** 2025-11-28

**Authors:** Hasan al-Hawasli, Mustafa Chawa, Bashar Younis

**Affiliations:** 1 Oubari Habboush Pharma, Damascus, Syria; 2 René Descartes Academy, Sorbonne University, Paris, France

**Keywords:** Growth hormone, Short stature, Syria, Middle east, Growth hormone deficiency, Idiopathic short stature, Small for gestational age, Turner syndrome

## Abstract

**Background:**

Growth hormone (GH) therapy is an effective option for short-stature children. In this study, we aimed to report our real-life experience of using GH treatment in Syria by comparing its effects in four different indications: growth hormone deficiency (GHD), idiopathic short stature (ISS), small for gestational age (SGA), and Turner syndrome (TS).

**Methods:**

We evaluated retrospectively the anthropometric data of 200 short-stature children who started therapy between 4 and 9 years and were treated between Jan-2018 and Jun-2023. Auxological parameters included growth velocity (GV), height (Ht), and bone age (BA).

**Results:**

After four years of GH therapy, all groups showed a significant increase in GVSDS and HtSDS (*p* < 0.00 for both). GHD revealed the most improvement (mean ΔGVSDS: 3.43) versus those with other indications (ISS: 2.39, SGA: 2.54, TS: 1.77). The mean ΔHtSDS observed ranged from a minimum of 0.82 in children with TS, 1.21 in SGA, and 1.48 in ISS to a maximum of 2.06 in GHD. The mean bone age-chronological age was − 1.59 at baseline and − 1.67 after 4 years (*p* = 0.18). The highest percentage of improvement in GVSDS and HtSDS was during the first year of treatment (for all groups, *p* < 0.00).

**Conclusions:**

For Syrian children with GHD, ISS, SGA, and TS, GH therapy provides an efficient treatment choice. GH therapy was most effective in those with GHD and during the first year of treatment. These results support the use of GH therapy for Syrian short-stature children.

**Clinical trial number:**

Not applicable.

## Introduction

Growth is a continual process; it is not linear. Both females and males go through three separate stages of growth: infancy, childhood, and pubertal. They experience these phases in a comparable way, although what grows and when differs for both genders, especially throughout puberty. Although linear growth is relatively rapid during the early stage of development, it thereafter slows down, resulting in an overall growth of 30 to 35 cm. Growing linearly in childhood occurs at a fairly steady rate. The majority of kids develop at the following rates: From 4 to 6 years, there is a growth rate of 5 to 8.5 cm/yr; from 6 years to puberty, it is 4 to 6 cm/yr for males and 4.5 to 6.5 cm/yr for females. Growth spurts (8 to 14 cm/yr) happen throughout puberty or adolescence as a result of the combined impacts of increased secretion of gonadal steroids and growth hormone (GH) [1].

Short stature (SS)is a public health problem that, by definition, affects 2.5% of the population around the world [2], it is regarded as one of the most frequent reasons for referrals to pediatric endocrinologists, second only to diabetes [3]. The evaluation of a child with SS must include an assessment of height velocity; this involves taking serial measurements over a period of at least 6 to 12 months to determine if the child’s height velocity is within the normal range or if it gradually deviates from it [1]. Additionally, radiographs of the hands and wrists must be taken to evaluate the child’s bone age as part of the diagnostic process for a short stature [1].

Growth hormone plays a critical role in the growth process. In the United States and Canada, cadaveric GH had been used for almost 30 years by 1985, either for research or therapeutic purposes. For the treatment of pediatric growth hormone deficiency (GHD), the FDA first approved recombinant human growth hormone (rhGH) in 1985 [4].

Which it later also approved for other indications such as Turner syndrome(TS) (1997), short children born small for gestational age(SGA) (2001), and idiopathic short stature (ISS) (2003) [4]. GH use in the U.S. pediatric population almost tripled from 2001–2016 [5].

From the healthcare providers’ perspective and among several indications approved for GH treatment by the Syrian Ministry of Health [6], GHD, ISS, TS, and SGA are the most frequent conditions for referral for GH treatment [7].

In Syria, due to the recent crisis it went through, the difficult economic situation, limited treatment options, and weak purchasing power, doctors are under pressure to prescribe high-cost medications. However, there is limited data on the real-world height outcomes of GH treatment in Syria.

The global literature provides a general framework for understanding GH therapy and its efficacy in different indications [8–11], but it is not a substitute for local evidence. The Syrian population exists at the intersection of unique genetic, environmental, and socio-economic factors that are not replicated elsewhere. Therefore, this study is not merely a repetition of global research; it is a necessary step to generate the specific, actionable evidence required to optimize care, guide policy, and ultimately improve the health outcomes of Syrian children with growth disorders. By providing a comparative analysis of treatment responses across four distinct pediatric indications, this study offers valuable real-world insights into the differential efficacy of growth hormone therapy, thereby informing clinical decision-making and resource allocation in a setting with limited healthcare resources. In this study, we aimed to report our real-life experience of using GH treatment in Syria by comparing its effects in four different indications.

## Methods

This retrospective study was performed by reviewing the medical records between January 2018 and June 2023 of all patients who started therapy between 4 and 9 years and were followed up regularly at the Chawa endocrinology, diabetes, and metabolism clinic. The Chawa clinic has the largest number of GH patients in a private endocrinologist clinic in Syria. Data were extracted from medical records that contained all required information; no imputation for missing data was performed. We included children with short stature (defined as height standard deviation score (HtSDS) < -2 for the same sex and chronologic age) [12] who received growth hormone treatment (Growtropin^®^, Dong-A, Korea) for GHD, ISS, SGA, or TS for ≥ 1 year.

Patients were categorized according to the etiology of short stature to four groups, namely: Growth Hormone Deficiency (GHD), Idiopathic Short Stature (ISS), Small for Gestational Age (SGA), and Turner Syndrome (TS).

Patients were monitored every three months during the duration of treatment. The doses of GH used were the product’s prescribing information intended daily dose (GHD: 0.6 IU (0.2 mg)/Kg/w, ISS: 1.1 IU (0.37 mg)/Kg/w, SGA: 1.44 IU (0.48 mg)/Kg/w, TS: 0.98 IU (0.33 mg)/Kg/w).

GHD was defined as a growth hormone level < 10 µg/L on two growth stimulation tests [13]. ISS was defined as having a height of 2 SDS < mean height for age, sex and population group, and in whom no identifiable disorder is present [13]. Small for gestational age (SGA) was defined as a birth weight and/or length < − 2 SDS and lack of catch-up growth after 4 years of life [12]. TS was diagnosed on the basis of a karyotype with complete loss of genetic material on the second sex chromosome [14].

Bone age assessments, as performed by a specialist physician, and adherence data, as documented in treating physician notes, were extracted from the medical records.

**Fig. 1:**
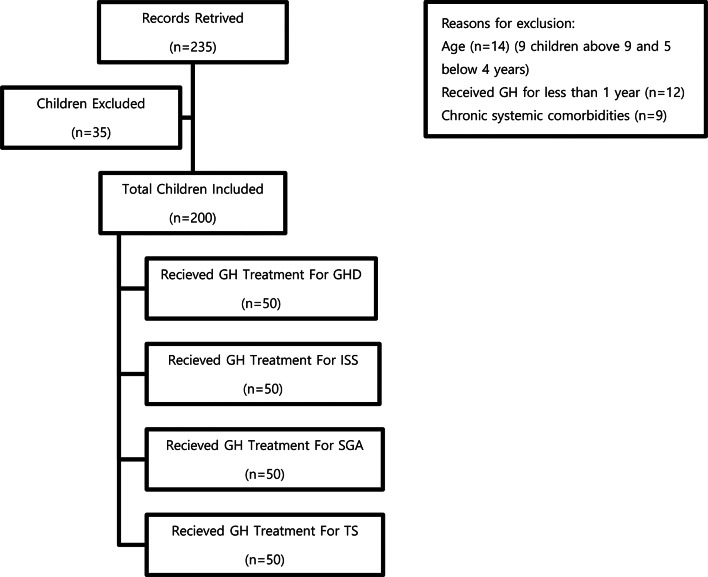
Study flow chart

Patients were excluded from the study if they presented with any of the following: chronic systemic comorbidities; syndromic or familial causes of short stature; evidence of pubertal onset (Tanner stage ≥ 2); or a history of treatment with medications known to influence growth (e.g., glucocorticoids, gonadotropin-releasing hormone analogs, aromatase inhibitors, or insulin-like growth factor-I). The study flow chart is presented in Fig. 1.

According to legislation in Syria, participant informed consent was unnecessary because of the study’s retrospective nature, anonymized recorded clinical data, and the impossibility of identifying participants directly or through identifiers in study results.

### Statistical analysis

Data were analyzed using SPSS, version 29.0 (IBM Inc, Armonk, NY, USA). Continuous variables were reported as mean ± SD, and categorial variables were reported as frequency and proportion. The normality of the data was checked using the Shapiro-Wilk test. Statistical significance was considered at a P-value ≤ 0.05. Descriptive analysis was performed to identify the baseline characteristics of study subjects.

Paired t-test was used to compare data after versus before GH treatment in each group. Mann-Whitney test was applied to compare the differences of numerical variables between the groups.

## Results

**Fig. 2:**
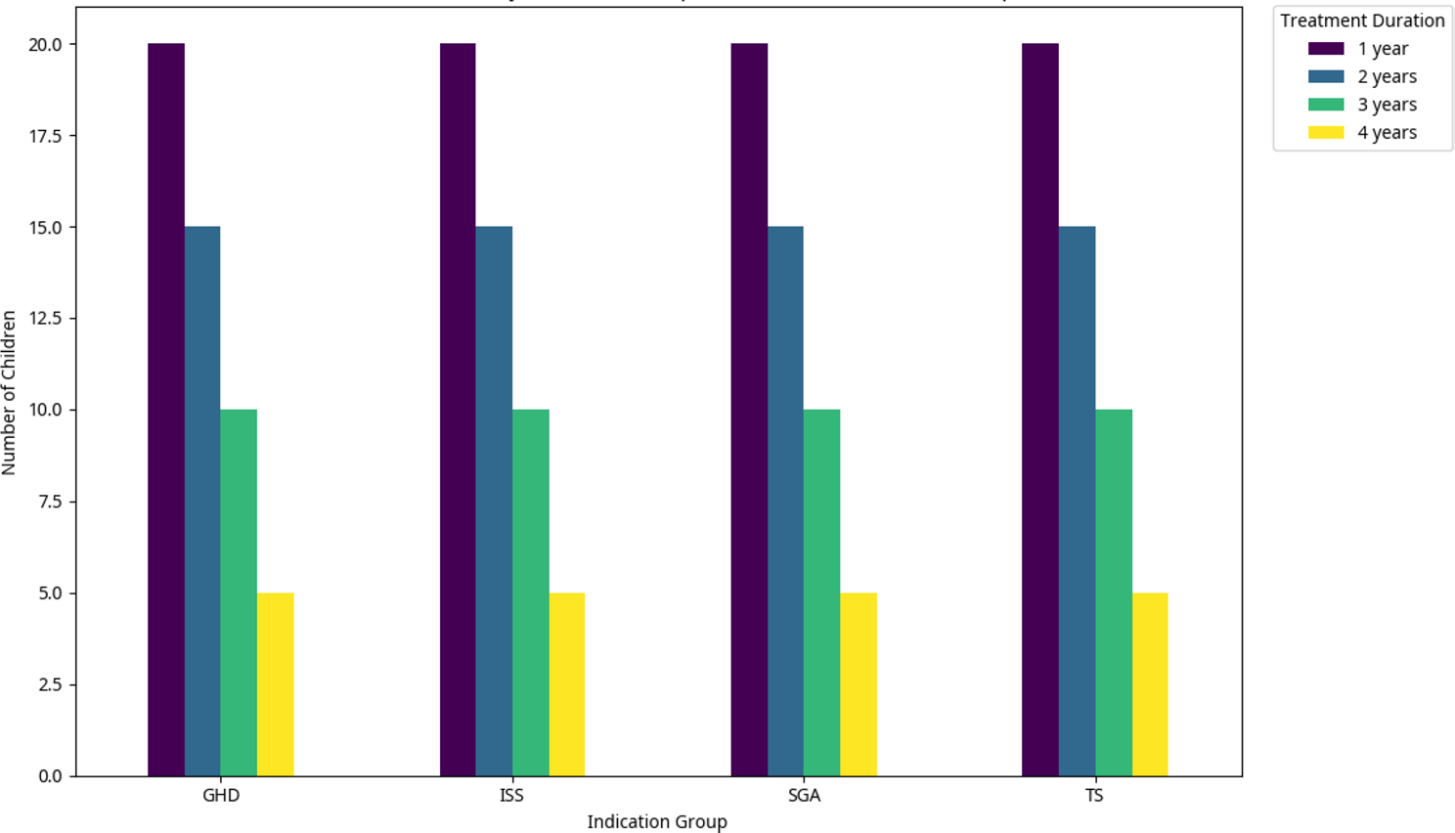
The distribution of children receiving GH treatment based on indication group and treatment duration. The distribution of children (*n* = 200) receiving GH treatment based on indication group (*n* = 50 for each) and treatment duration. GHD: growth hormone deficiency; ISS: idiopathic short stature; SGA: small for gestational age; TS: Turner syndrome

A total of 200 children who started GH treatment between the ages of 4 and 9 years were included in the analysis. The age groups were [4–5] years (10.0%), [5–6] years (19.5%), [6–7] years (22.5%), [7–8] years (21.0%), and [8–9] years (27.0%). 50 pediatric patients in each indication group received treatment for at least a year. Among these patients, 20 children received treatment for one year, 15 children received treatment for two years, 10 children received treatment for three years, and 5 children received treatment for four years. The distribution of children receiving GH treatment based on indication group and treatment duration is presented in Fig. 2.

There was no significant difference in baseline characteristics between females and males (all baseline characteristics, *p* > 0.05), as presented in Table 1.

**Table 1:** Baseline characteristics according to gender

Variable/mean (SD)	ALL	Male	Female	*P*-Value
AGE (year)	6.50 (1.46)	6.34 (1.45)	6.54 (1.47)	0.317
95% Confidence interval	(6.31,6.72)	(6.05,6.70)	(6.34,6.87)	
Body Weight (kg)	14.27 (2.45)	14.41 (2.66)	14.17 (2.32)	0.689
95% Confidence interval	(13.92,14.61)	(13.82,15.00)	(13.74,14.58)	
BMI	14.24 (1.17)	14.36 (1.04)	14.16 (1.24)	0.069
95% Confidence interval	(14.06,14.39)	(14.13,14.59)	(13.91,14.36)	
Height (cm)	99.82 (7.76)	99.78 (7.73)	99.85 (7.81)	0.902
95% Confidence interval	(98.76,100.93)	(98.05,101.50)	(98.47,101.31)	
Height SDS	-3.22 (0.51)	-3.20 (0.49)	-3.23 (0.52)	0.690
95% Confidence interval	(-3.29, -3.15)	(-3.31, -3.09)	(-3.33, -3.14)	
Growth velocity (cm/y)	3.06 (0.61)	3.11 (0.71)	3.02 (0.54)	0.095
95% Confidence interval	(2.97,3.14)	(2.95,3.27)	(2.92,3.11)	
Bone Age (year)	4.91 (1.54)	4.78 (1.55)	4.99 (1.54)	0.347
95% Confidence interval	(4.69,5.13)	(4.44,5.13)	(4.72,5.28)	

GHD: growth hormone deficiency; ISS: idiopathic short stature; SGA: small for gestational age; TS: Turner syndrome; BMI: body mass index, SDS: standard deviation score; GV: growth velocity; BA: bone age; CA: chronological age

The baseline anthropometric characteristics of all the patients according to indication are summarized in Table 2. After excluding patients with TS from the analysis, no significant difference was found in the gender of those who received GH treatment (*p* = 0.974).

**Table 2:** Baseline anthropometric characteristics according to indication

Variable	ALL	GHD	ISS	SGA	TS	*P*-value
Gender	**(** ***n*** ** = 200)**	**(** ***n*** ** = 50)**	**(** ***n*** ** = 50)**	**(** ***n*** ** = 50)**	**(** ***n*** ** = 50)**	
Male	80 (40%)	26 (52%)	27 (54%)	27 (54%)	0 (0%)	0.974*
Female	120 (60%)	24 (48%)	23 (46%)	23 (46%)	50 (100%)	
Age (y)	6.50 (1.46)	6.23 (1.52)	6.76 (1.42)	6.37 (1.41)	6.49 (1.49)	0.310
95% CI	(6.30, 6.71)	(5.84, 6.70)	(6.40, 7.21)	(6.01, 6.81)	(6.11, 6.96)	
Body Weight (kg)	14.27 (2.45)	13.86 (2.87)	15.10 (2.25)	13.46 (2.31)	14.64 (2.03)	0.003
95% CI	(13.92, 14.61)	(13.04, 14.68)	(14.46, 15.74)	(12.80, 14.12)	(14.06, 15.22)	
BMI	14.24 (1.17)	14.27 (1.20)	14.00 (0.89)	13.70 (1.03)	14.97 (1.15)	0.000
95% CI	(14.07,14.40)	(13.93,14.61)	(13.75,14.25)	(13.41,14.00)	(14.64,15.30)	
Height (cm)	99.82 (7.76)	98.04 (8.28)	103.60 (7.28)	98.76 (7.04)	98.82 (7.26)	0.001
95% CI	(98.74,100.90)	(95.69,100.39)	(101.59,105.73)	(96.76,100.76)	(96.75,100.89)	
Height SDS	-3.22 (0.51)	-3.36 (0.53)	-2.79 (0.40)	-3.35 (0.37)	-3.36 (0.47)	0.000
95% CI	(-3.29, -3.15)	(-3.51, -3.21)	(-2.91, -2.68)	(-3.46, -3.25)	(-3.50, -3.23)	
Growth velocity (cm/y)	3.05 (0.61)	2.96 (0.69)	3.16 (0.61)	3.24 (0.51)	2.85 (0.54)	0.006
95% CI	(2.97,3.14)	(2.76,3.16)	(2.98,3.34)	(3.09,3.39)	(2.70,3.01)	
Bone Age	4.91 (1.54)	4.01 (1.31)	5.63 (1.40)	4.80 (1.54)	5.18 (1.46)	0.000
95% CI	(4.69,5.12)	(3.64,4.38)	(5.23,6.03)	(4.36,5.24)	(4.76,5.60)	

GHD: growth hormone deficiency; ISS: idiopathic short stature; SGA: small for gestational age; TS: Turner syndrome; BMI: body mass index, SDS: standard deviation score; GV: growth velocity; BA: bone age; CA: chronological age; CI: confidence interval. Values are numbers (%) or means (SD) as appropriate

*: After excluding patients with TS from the analysis

The mean age across all groups is approximately 6.5 years, with no statistically significant difference between the subtypes (P-value = 0.31). In contrast, there are notable differences in body weight among the groups (P-value = 0.003), with SGA patients having the lowest mean weight (13.46 kg).

Height also differs significantly (P-value = 0.001). ISS patients are the tallest on average (103.6 cm), while GHD patients are the shortest (98.04 cm). The mean heights of SGA (98.76 cm) and TS (98.82 cm) patients are similar and slightly greater than that of GHD patients.

Assessing the severity of short stature using height standard deviation score (SDS) reveals that ISS patients are the least affected (mean SDS: -2.79). GHD and TS patients have the most severe short stature (mean SDS: -3.36 for both), closely followed by SGA patients (mean SDS: -3.35).

Finally, growth velocity also varies, with SGA patients having the highest rate (3.24 cm/y), followed by ISS (3.16 cm/y). GHD (2.96 cm/y) and TS (2.85 cm/y) patients have the lowest growth velocities.

### Changes in growth velocity (Figs. 3 and 4)

After 4 years of therapy, all children showed significant improvement in GV patterns (mean 3.06 to 5.20 cm/y)(*p* = 0.0001). Patients achieved the best GV improvement in the 1st year (means: GHD: 6.30 cm/y, ISS: 5.70 cm/y, SGA: 5.55 cm/y, TS: 4.69 cm/y)(all indications; *p < 0.000), which later dropped to within the normal growth velocity range for their age.

**Fig. 3:**
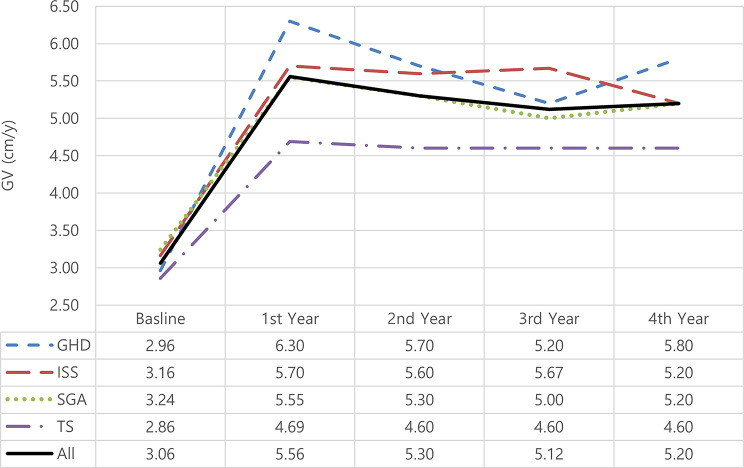
Change in growth velocity (cm/y) among treated patients according to years of treatment and indication. Sample sizes for each indication are *n* = 50 at baseline. GHD: growth hormone deficiency; ISS: idiopathic short stature; SGA: small for gestational age; TS: Turner syndrome; GV: growth velocity. *p < 0.000

This significant increase in growth velocity after 4 years corresponded to a change in GV-SDS mean from − 2.41 to 0.12(*p* = 0.000), GHD revealed the most final improvement (mean ΔGVSDS: 3.43) versus those with other indications (mean ΔGVSDS: ISS: 2.39, SGA: 2.54, TS: 1.77).

**Fig. 4:**
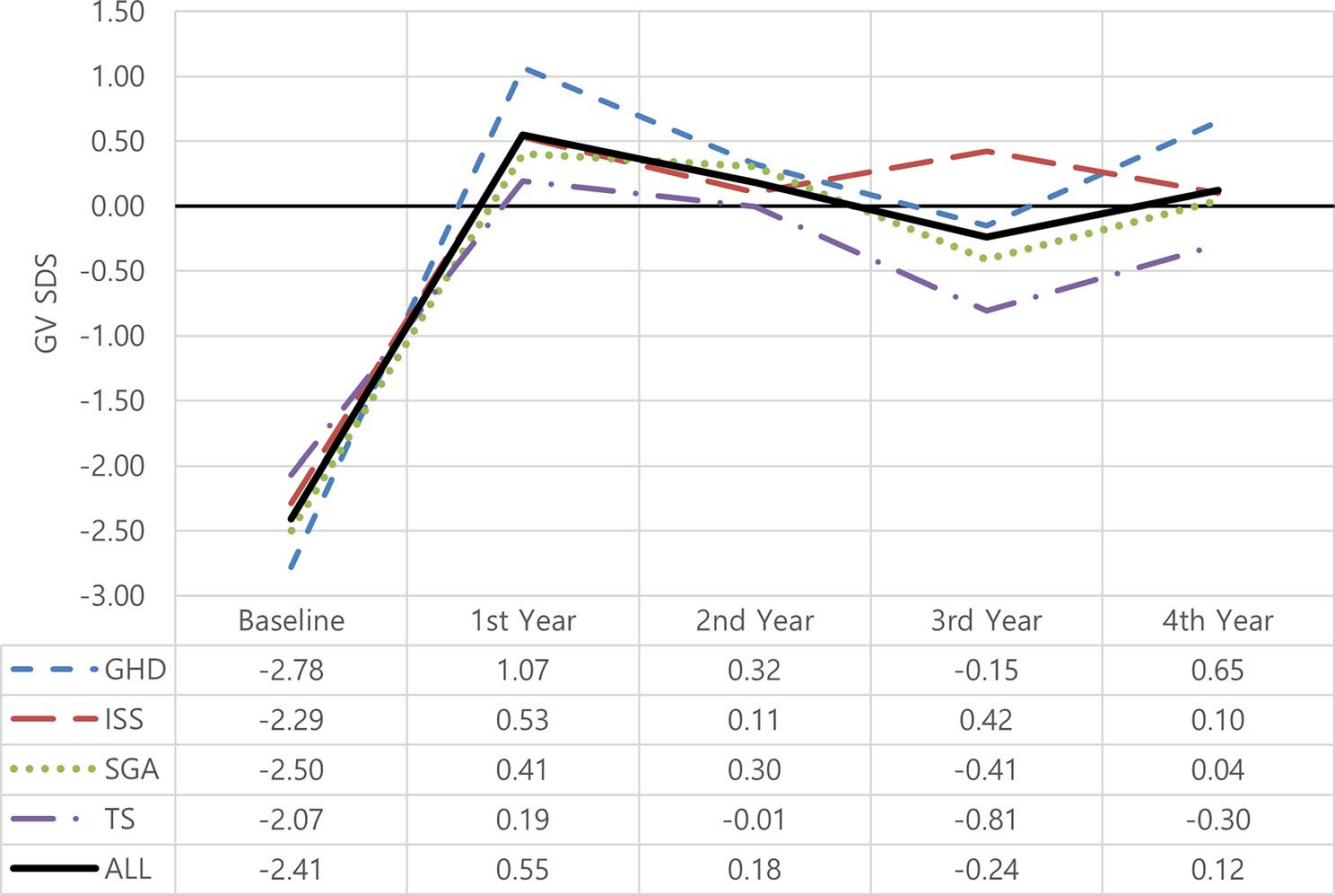
ΔGVSDS among treated patients according to years of treatment and indication. Sample sizes for each indication are *n* = 50 at baseline. GHD: growth hormone deficiency; ISS: idiopathic short stature; SGA: small for gestational age; TS: Turner syndrome; SDS: standard deviation score; GV: growth velocity

### Change in HtSDS (Fig. 5)

After 4 years of therapy with GH, all patients achieved a significant height gain (all indications; *p* < 0.00), and HtSDS was improved. The pattern of increases in GV values was reflected in the simultaneous correction of HtSDS. The highest percentage of correction was during the first year of treatment, and after 4 years, it reached an average of about 1.4 ΔHtSDS (the mean height SDS change observed ranged from a minimum of 0.82 in children with TS, 1.21 in SGA, 1.48 in ISS, to a maximum of 2.06 in children with GHD).

**Fig. 5:**
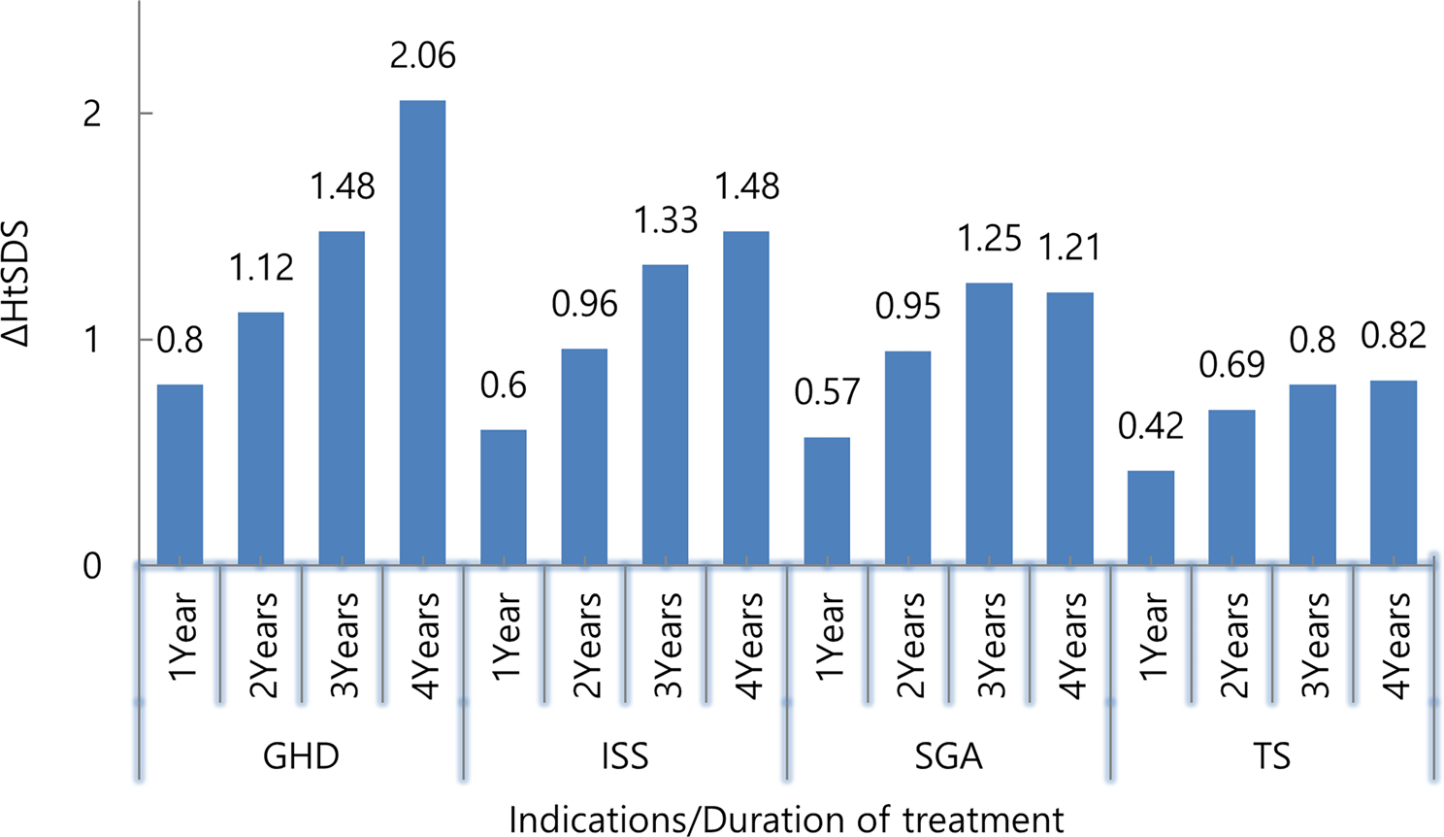
Change in height SDS (ΔHtSDS) among treated patients according to years of treatment and indication. Sample sizes for each indication are *n* = 50 at baseline. GHD: growth hormone deficiency, ISS: idiopathic short stature; SGA: small for gestational age; TS: Turner syndrome; BMI: body mass index; HtSDS: height standard deviation score

### Change in BA-CA (Fig. 6)

The mean (SD) bone age (years) was 4.91 (1.54) at baseline and 7.33 (0.89) after 4 years. The mean (SD) bone age-chronological age was − 1.59 (0.66) at baseline and − 1.67 (0.65) after 4 years (*p* = 0.18).

However, there was no statistically significant difference in all indication groups in ΔBA-CA between treatment initiation and 4 years after it.

**Fig. 6:**
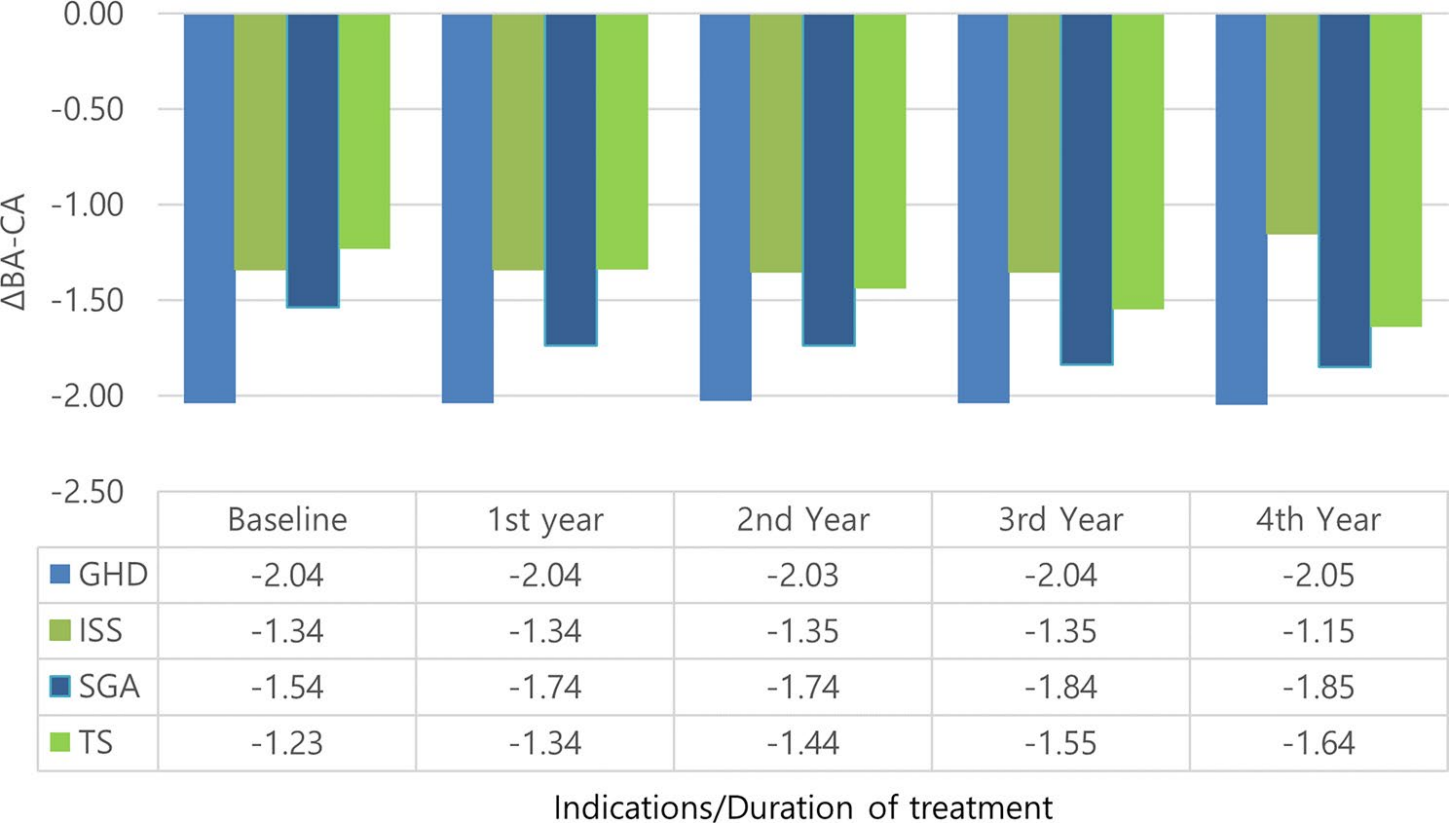
Change in BA-CA among treated patients according to years of treatment and indication. Sample sizes for each indication are *n* = 50 at baseline. GHD: growth hormone deficiency, ISS: idiopathic short stature, SGA: small for gestational age; TS: Turner syndrome; BA: bone age; CA: chronological age

### Predictors of growth outcomes in GH therapy (Table 3)

In this study, we evaluated the baseline factors contributing to the ΔGVSDS, ΔHtSDS, and ΔBA-CA after 4 years of GH therapy (Table 3). In the multivariate linear regression analysis of our findings, the duration of treatment (β = −0.622, *p* = 0.005) was significantly associated with ΔGVSDS.

Moreover, age at GH initiation, duration of treatment, and indication were identified as the variables contributing to the total variability in ΔHtSDS improvement (β = 0.163, *p* = 0.000, β = −0.287, *p* = 0.000, β = −0.175, *p* = 0.000) respectively.

By linear regression modeling, indication was the only variable contributing to the ΔBA-CA (β = −0.069, *p* = 0.001).

**Table 3:** Baseline variables contribution in outcomes improvement

Variable	ΔGVSDS		ΔHtSDS		ΔBA-CA	
	β	*P*-Value	β	*P*-Value	β	*P*-Value
Intercept	-28.244	0.182	2.908	0.163	-1.541	0.512
Age at GH initiation	-0.424	0.301	0.163	0.000	0.020	0.667
Indications	-0.339	0.067	-0.175	0.000	-0.069	0.001
Duration of treatment	-0.622	0.005	0.287	0.000	-0.024	0.334

GVSDS: growth velocity standard deviation score; HtSDS: height standard deviation score; BA: bone age; CA: chronological age

## Discussion

This is the first study to look at the long-term effects of GH therapy in Syria. No previous studies have been conducted in Syrian children regarding the use of GH or comparing its effects among different indications. All that was found was limited to some case reports [15–17] or an etiology classification study [18]. Also, most countries rely on WHO, CDC, or French growth charts [7, 19], and while growth charts were issued in some neighboring countries several decades ago (e.g., KSA in 2007) [20], we herein adhere to the recently nationally approved growth reference charts for the Syrian population [21]. Moreover, this study conforms with Syrian guidelines for GH therapy [6], whereas most of the below-reviewed studies adhered to international guidelines; little of them were based on national guidelines (e.g., Saudi Arabia) [22].

Accurate assessment of growth velocity is a critical component in the diagnostic evaluation of growth retardation [23]. However, response to GH therapy is a highly variable outcome influenced by multiple factors, including the dose of GH, young age at initiation [24], the duration of treatment, distance between the patient’s height SDS and mid-parental height SDS [20], indication, severity of disease, comorbidities and adherence [22], and the first-year response to GH treatment [25, 26].

Bone age assessment also plays a vital role in diagnosing short stature, as it helps to differentiate between conditions associated with delayed skeletal maturation and those with a normal or accelerated bone age relative to chronological age (CA) [1].

In some countries, children with short stature are diagnosed at a relatively older age, which might influence the benefits of early treatment [27]. Early screening for growth failure and subsequent referral to a pediatric endocrinology service are very important to prevent undue delays in the diagnosis and management of treatable causes of short stature [27]. However, different from patients reported in the literature, the patients’ population in this study received GH therapy at an earlier age for all indications (6.50 years) compared to the French SAGhE Study (11.0 years) and PATRO Children study Italian cohort (10.0 years). Also, GHD children in this study started GH therapy at a mean age of 6.23 years, compared to 10.2 years from the International ANSWER Registry and 9.2 years from the Pfizer International Growth Database (KIGS) [28]. In contrast, children with Idiopathic Short Stature (ISS) were diagnosed at a slightly older mean age (6.76 years) compared to the cohort average. This is consistent with the diagnostic pathway for ISS, which is often one of exclusion, potentially requiring a period of observation before confirmation.

### GHD cohort

In agreement with previous studies [24, 29–31], and as shown in Figs. (4, 5, and 6), ΔGVSDS, ΔHtSDS, and ΔBA-CA were higher in patients with GHD than in patients with other indications.

GV is the most common parameter measured to assess growth response in the first year of treatment, followed by ΔHSDS [22]. In this study, the change in mean GVSDS was 3.43 after 4 years of therapy. The mean ΔGVSDS was higher in the first year of treatment (3.85) than it was in the subsequent years (-0.75, -0.47, 0.80), respectively, supporting other published studies’ findings [26, 32–34] that the first year of treatment is when patients respond most favorably.

**Fig. 7:**
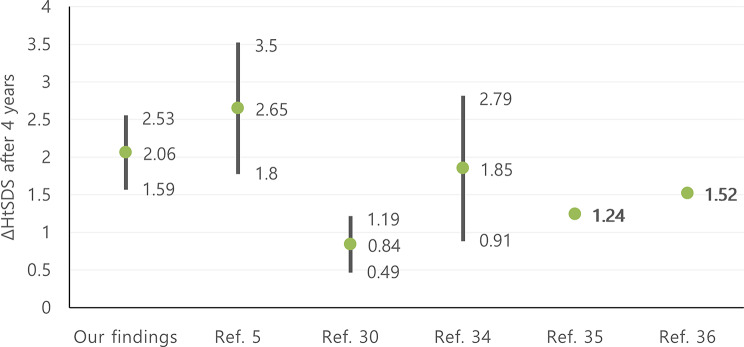
Comparison of ΔHtSDS in GHD cohort after 4 years, in our findings with previous studies. HtSDS: height standard deviation score

According to our results, GH continued to have a positive impact on HtSDS gain for up to four years (*p* = 0.000). As presented in Fig. 7, ΔHtSDS after 4 years of therapy was 2.06 ± 0.47 and this is compatible with previous studies (between 1.8–3.5) [5], (0.84 ± 0.35) [30], (1.85 ± 0.94) [34], (mean 1.24) [35], (mean 1.52) [36].

The mean ΔHtSDS was also higher in the first year of treatment (0.8 ± 0.57) than it was in the subsequent years (means: 0.53, 0.39, 0.51) respectively. These results are consistent with previous studies that reported mean ΔHtSDS after 1 year: KIGS analyses (1.01) [37], sub-analysis of PATRO children (0.57) [35], Al-Abdulrazzaq et al. [28] (median: 0.57), H. Lim [33] (0.84), L. Hou [29] (0.74), and S. W. Yang et al. [38] (0.6).

### ISS cohort

The same patterns of results were observed in this group, in which Δ change decreased gradually (ΔGVSDS, ΔHtSDS, and ΔBA-CA: after 1 year: 2.82, 0.6, -0.01, after 4 years: 2.39, 1.48, -0.02), respectively. This is still in line with data reported from previous studies [29] who reported ΔHtSDS (mean: 1.56) after 3 years; Al-Abdulrazzaq et al. [28] also observed ΔHtSDS (0.74) after 1 year; J. Kim et al. [39] observed ΔHtSDS (0.63) after 0.5 year; and Y.-Q. Ying et al. [40] reported ΔHtSDS (0.44 after 0.5 year and 1.95 after 4 years). While in Al Shaikh et al. [41], ΔHtSDS after 3 years was only 0.65, i.e., lower than those reported here. However, direct comparisons between our study and the others are difficult here due to differences in study duration and sample size.

### SGA cohort

**Fig. 8:**
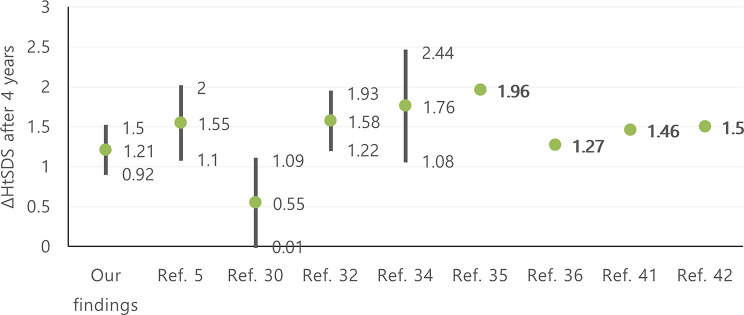
Comparison of ΔHtSDS in SGA cohort after 4 years, in our findings with previous studies. HtSDS: height standard deviation score

As presented in Fig. 8, the final change after 4 years in height SD was 1.21 ± 0.29 and this is compatible with previous studies: ΔHtSDS (between 1.1–2) [5], (0.55 ± 0.54) [30], (between 1.22–1.93) [32], (1.76 ± 0.68) [34], (mean: 1.96) [35], (mean: 1.27) [36], (mean: 1.46) [41], (mean: 1.5) [42].

Related to the response to treatment in the first year, our results (ΔHtSDS 0.57 ± 0.38) are consistent with those of these earlier studies (ΔHtSDS between 0.23–0.63) [32], sub-analysis of PATRO Children(0.48) [35].

### TS cohort

Short stature is the most consistent characteristic feature of TS. Untreated females with TS achieve an average adult height of ~ 140 cm [43]. In our results, ΔHtSDS was 0.82 ± 0.43 after 4 years, and this is compatible with previous studies: 0.33 after 9.4 years [30], 1 after 7 years [41], 1 after 5 years [34], 0.86 after 4 years [36], 0.7 after 1 year [43], 0.59 ± 0.34 after 1 year [44]. 

### Growth outcomes of GH therapy in Syrian children

In our study, the growth effects for most indications were among the highest reported in the literature. This can be related to multiple factors: adherence to the indicated dose, early initiation of rhGH therapy, and extended duration of treatment.

However, direct comparisons between our study and other observational studies/registries are difficult due to differences in study design, population baseline characteristics, age groups, publication bias, and other confounding factors. As well as comparison studies in different etiologies are not routinely performed, and sample sizes are not homogeneous.

This study has several limitations that must be acknowledged. Firstly, the absence of an untreated control group means that the observed improvements in growth parameters represent within-group changes over time. This study has also the inherent limitations of a retrospective design. Specifically, no formal sample size calculation or power analysis was conducted a priori, as the analysis included all available patients from the clinic’s records who met the inclusion criteria over the defined period.

It’s a single-center design, while reflective of a major referral practice, inherently relies on medical records, which led to challenges such as missing patient safety data and a lack of detailed, objective measures of treatment adherence.

The limited number of patients who continued treatment for a long period of time and the absence of adjustments for potential confounding factors (Socioeconomic Status, Nutritional Status, Parental Height, etc.) limit the robustness of direct comparisons between these groups; future studies with multivariate analysis are warranted to confirm these findings. Furthermore, as the study was conducted in a private practice setting, the patient population may be more likely to adhere to treatment and maintain follow-up than the general Syrian population, potentially introducing a selection bias that favors better outcomes. These factors should be considered when generalizing the results. Finally, considering the lack of research on GH therapy in Syria, the study results were compared with previous studies in other countries where the average height may differ.

However, our study has several strengths. First, this study was an observational study of real-life use of GH rather than a controlled trial of therapy, which cannot be ideally translated into everyday clinical practice. Second, we reported HtSDS changes based on Syrian growth charts data rather than global charts to allow for a real comparison of growth hormone effects [12]. Third, we provided a comparison among four different approved indications for rhGH therapy from a Syrian perspective. Finally, the focus was on patients in the most difficult stage of treatment, the stage in which the androgen axis is not yet fully developed, so it does not interfere with the growth hormone’s effects, and the role of growth hormone is evident.

A key question for future research is whether growth patterns differ significantly between patients with peak GH levels below 7 ng/mL and those with levels between 7 and 10 ng/mL, given that present data preclude a consensus on an exact GH cut-off value for confirming GHD [13].

The baseline characteristics and GH therapy outcomes of 200 Syrian children with GHD, ISS, SGA, and TS were examined using real-world retrospective data with a minimum follow-up of 12 months. While randomized controlled trials have shown the short-term efficacy and safety of GH therapy in the studied indications [38, 39, 43, 45], long-term retrospective cohort studies like this one balance clinical trials by providing data on actual patient characteristics and outcomes.

## Conclusion

This study showed that GH treatment was associated with increases in both GVSDS and HtSDS in all 4 groups and provided additional evidence for the long-term effectiveness of GH therapy for Syrian short-stature children. GH therapy was most effective in increasing height SDS, particularly in those with GHD, and during the first year of treatment. The duration of GH treatment was significantly associated with improved growth velocity. Age at initiation of GH therapy and duration of treatment played a role in improving height SDS. The difference between bone age and chronological age did not change significantly after 4 years of therapy. Despite the challenges of treating children aged 4–9 years with GH therapy, it should not be neglected at this stage, and there should be no delay in initiating treatment for these children.

Overall, this study provides valuable information about the long-term effects of GH therapy in Syrian children with GHD, ISS, SGA, and TS.

## Data Availability

The datasets used and/or analyzed during the current study are available from the corresponding author on reasonable request.

## Abbreviations

BA: Bone age
BMI: Body mass index
CA: Chronological age
CDC: Centers for disease control and prevention
CI: Confidence interval
GH: Growth hormone
GHD: Growth hormone deficiency
GV: Growth velocity
GVSDS: Growth velocity standard deviation score
Ht: Height
HtSDS: Height standard deviation score
ISS: Idiopathic short stature
rhGH: recombinant human growth hormone
SDS: Standard deviation score
SGA: Small for gestational age
SS: Short stature
TS: Turner syndrome
WHO: World health organization
